# Investigating the Metabolism of Plants Germinated in Heavy Water, D_2_O, and H_2_^18^O-Enriched Media Using High-Resolution Mass Spectrometry

**DOI:** 10.3390/ijms242015396

**Published:** 2023-10-20

**Authors:** Sergey Osipenko, Anton Bashilov, Anna Vishnevskaya, Lidiia Rumiantseva, Anna Levashova, Anna Kovalenko, Boris Tupertsev, Albert Kireev, Eugene Nikolaev, Yury Kostyukevich

**Affiliations:** 1 Skolkovo Institute of Science and Technology, Bolshoy Boulevard 30, Bld. 1, 121205 Moscow, Russia; sergey.osipenko@skoltech.ru (S.O.); a.bashilov@skoltech.ru (A.B.); ai.vish@yandex.ru (A.V.); lidiia.rumiantseva@skoltech.ru (L.R.); a.levashova@skoltech.ru (A.L.); a.kovalenko@skoltech.ru (A.K.); b.tupertsev@skoltech.ru (B.T.); a.kireev@skoltech.ru (A.K.); e.nikolaev@skoltech.ru (E.N.); 2 Institute for Translational Medicine and Biotechnology, First Moscow State Medical University, 119991 Moscow, Russia

**Keywords:** mass spectrometry, garden cress, plant metabolomics, isotope exchange, H/D exchange, stable isotope labeling

## Abstract

Mass spectrometry has been an essential technique for the investigation of the metabolic pathways of living organisms since its appearance at the beginning of the 20th century. Due to its capability to resolve isotopically labeled species, it can be applied together with stable isotope tracers to reveal the transformation of particular biologically relevant molecules. However, low-resolution techniques, which were used for decades, had limited capabilities for untargeted metabolomics, especially when a large number of compounds are labelled simultaneously. Such untargeted studies may provide new information about metabolism and can be performed with high-resolution mass spectrometry. Here, we demonstrate the capabilities of high-resolution mass spectrometry to obtain insights on the metabolism of a model plant, *Lepidium sativum*, germinated in D_2_O and H_2_^18^O-enriched media. In particular, we demonstrated that in vivo labeling with heavy water helps to identify if a compound is being synthesized at a particular stage of germination or if it originates from seed content, and tandem mass spectrometry allows us to highlight the substructures with incorporated isotope labels. Additionally, we found in vivo labeling useful to distinguish between isomeric compounds with identical fragmentation patterns due to the differences in their formation rates that can be compared by the extent of heavy atom incorporation.

## 1. Introduction

Plants can produce a diverse set of metabolites, though only a small fraction of them are identified and organized into databases [1]. Untargeted plant metabolomics is the most promising approach to uncover new plant metabolites and obtain insights into metabolic pathways [2]. Advances in mass spectrometry instrumentation made possible the simultaneous detection of thousands of compounds in one analytical run, and the developed approaches for structure elucidation with mass spectrometry data in many cases allow us to correctly annotate primary and secondary metabolites [3,4,5,6,7,8].

To follow the biotransformation of a particular molecule involved in metabolism, stable isotope tracers can be used, though this requires advanced analytical techniques to resolve isotopologues. In vivo stable isotope labeling has found a broad application in mass spectrometry-based metabolomics studies [9,10,11,12,13,14,15,16] In vivo deuterium labelling was used to elucidate the biosynthesis of cytokinins [17,18] and the metabolism of indole-3-acetic acid [19]; deuterium and ^18^O-labeled choline were used to study betaine oxidative formation [20], while ^15^N-tracers [21] are valuable for investigating protein synthesis. The majority of studies, however, implement ^13^C (mainly in the form of ^13^CO_2_) labeling of plants [22,23,24,25,26] to follow carbon skeleton transformations in living cycles [27].

Besides the opportunity to highlight the routes of molecules in metabolic cycles, stable isotopes provide valuable techniques to elucidate chemical structures and discriminate between isobaric compounds [28]. Although such techniques are often deployed ex vivo, for example as in-solution [29,30,31] or in-source H/D [32,33,34,35] and ^16^O/^18^O exchange [36,37,38,39,40], it was shown that in vivo labelling may improve molecular formula assignment [41] and the identification of unknown flavonoids [42]. In vivo labeling is also applied to evaluate metabolic turnover rates in mammals [43,44,45] or rodents [46].

All plant metabolomics studies that involve isotope labelling can be roughly divided into two groups. The first group includes targeted studies, where specific molecules were labelled and their transformation was followed; the second group includes untargeted research using basic substrates, such as CO_2_, H_2_O and O_2_, to introduce isotopic labels to the metabolites. The latter requires a more complicated analytical approach as the label may appear in a wide range of biologically relevant molecules. To our surprise, the number of studies that implement an untargeted approach with D_2_O as the labelling source is very limited, and we could not find any mention of in vivo plant labelling for untargeted metabolomics with H_2_^18^O.

The use of heavy water (D_2_O and H_2_^18^O) as a heavy isotope source has the potential for simultaneously labeling large numbers of plant metabolites in a technically simple and cost-efficient way. Here, using the example of a model plant *Lepidium sativum* (garden cress), we implement in vivo plant labelling with D_2_O and H_2_^18^O to evaluate the capabilities of untargeted mass spectrometry-based metabolomics with stable isotope tracers.

## 2. Results

### 2.1. LC–HRMS Analysis of Labeled Plant Extracts

Garden cress was germinated with 1:1 mixtures of D_2_O and H_2_O and H_2_^18^O:H_2_O for 12 days in 12 mL vials with a total heavy water uptake of 1.5 mL. Mixing with H_2_O was essential for the D_2_O experiments, as pure deuterium oxide is toxic and prevents the growing of plants. This effect is well known and was observed in our preliminary experiments.

**Figure 1:**
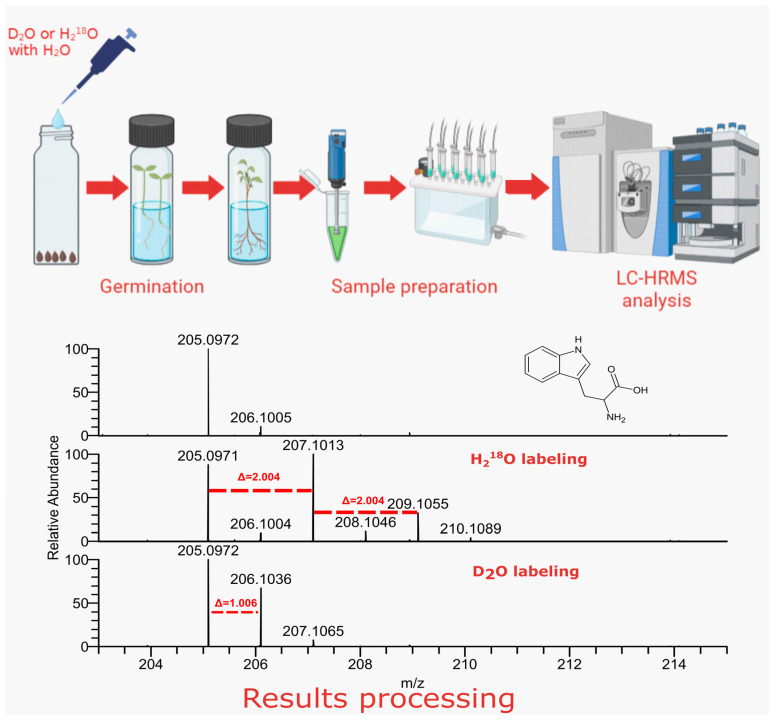
Overview of the proposed in vivo labeling approach. The spectra on the bottom correspond to tryptophan found in non-labeled *Lepidium sativum* extract and in the extracts of *Lepidium sativum*, germinated in D_2_O or H_2_^18^O water mixtures. Red dashed lines mark shifts in mass spectra, corresponding to heavy atom incorporation.

LC–HRMS analysis was performed in both positive and negative DDA modes; we managed to annotate a number of primary metabolites, such as amino acids, carbohydrates and organic acids, and secondary metabolites, including glucosinolates, cinnamic acid derivatives and flavonoids. For each annotated compound, we calculated the number of incorporated heavy atoms by counting additional peaks in the mass spectra of labeled plants with characteristic mass shifts of 1.0063 and 2.0042 for deuterium and ^18^O, respectively, as shown in Figure 1, with the example of tryptophan. Table 1 summarizes the annotated compounds with the calculated number of stable isotope labels. Annotation was performed via an MS/MS library search against the mzCloud and MassBank libraries. The results of a supplementary GC–MS analysis are given in the Table S1.

### 2.2. The Contribution of the Isotope Exchange Reaction

**Figure 2:**
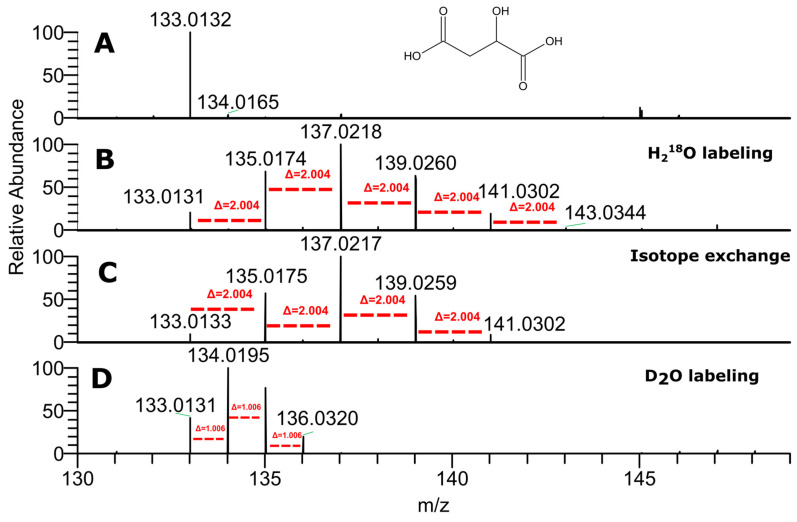
Mass spectra of malic acid from unlabeled plant extract (**A**), plant labeled with H_2_^18^O (**B**), pure malic acid after 21-day incubation with H_2_^18^O (**C**) and from D_2_O plant labeled extract (**D**) in negative ion mode. Red dashed lines indicate shifts in the mass spectrum, corresponding to heavy atom incorporation.

For malic acid, it was demonstrated that oxygen-^18^O incorporation may not only be the result of an exchange reaction but also occurs during biosynthesis. Indeed, in the mass spectrum of malic acid from ^18^O-labeled garden cress, we observed five inclusions of the ^18^O atom (Figure 2A,B). Four exchanges of carboxyl oxygen atoms were expected, but we had to verify if the atom in the hydroxyl group was introduced due to exchange. For verification, we incubated pure malic acid (1 mg/mL) with H_2_^18^O for 21 days at room temperature; however, only four exchanges were detected (Figure 2C). Therefore, at least one of the five oxygen atoms in malic acid were incorporated into the malic acid molecule as the result of biosynthesis. The mass spectrum of malate from the D_2_O labeled plant (Figure 2D) demonstrates the inclusion of three deuterium atoms, which indicates that hydrogen atoms from water are also involved in its biosynthesis.

In vivo labeling of primary metabolites. Although the separation method used, based on reversed phase HPLC, is not suitable for polar compounds, which include most of the primary metabolites, we detected amino acids, organic acids, vitamins and carbohydrates. For all detected amino acids, at least two oxygen-^18^O atoms were observed, most likely due to the isotope exchange reaction. For amino acids with additional oxygen-containing functional groups (glutamine, asparagine, tyrosine and threonine), we detected molecules where all oxygen atoms were replaced by ^18^O, which reflects the amino acid synthesis. However, for the deuterated plants, the extent of label incorporation was different for various amino acids. For example, for tryptophan, only one deuterium atom was detected, while for phenylalanine we observed an ion with 5 deuterium atoms. Both compounds are produced in the shikimate metabolic pathway; however, the deuterium label in tryptophan is located in the aliphatic chain, while for phenylalanine, labeling of the aromatic ring is observed according to the MS/MS data. A possible explanation is that tryptophan precursors (anthranilate or indole) originated from seeds, while phenylalanine predecessors were also formed during germination.

**Figure 3:**
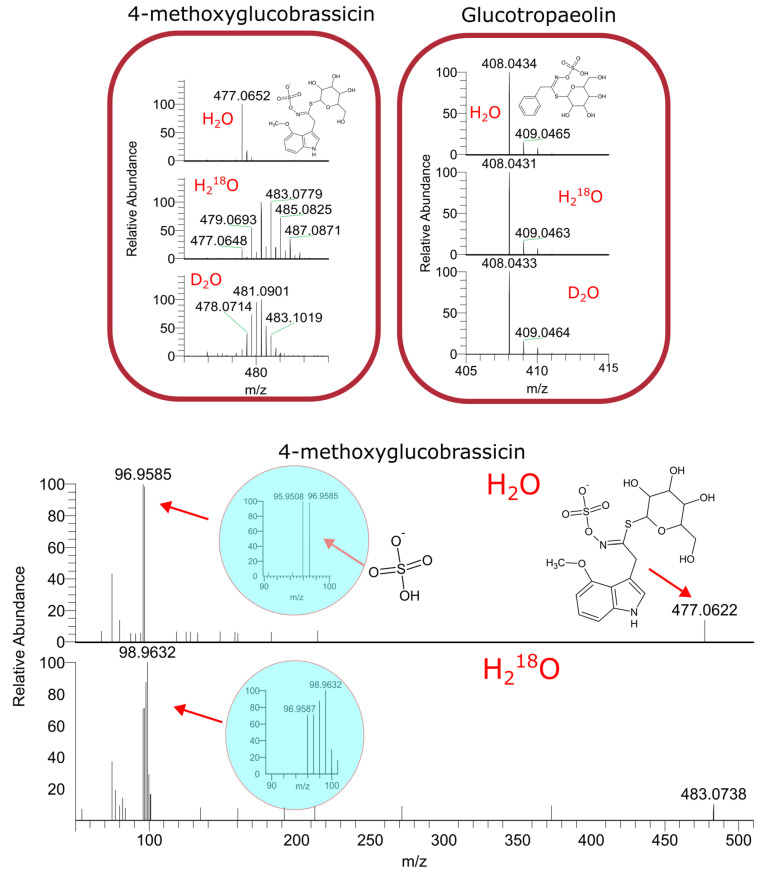
Mass spectra of glucotropaeolin (**right**) and 4-methoxyglucobrassicin (**left**) from isotopically labeled plants. On the bottom figure, the MS/MS of labeled 4-methoxyglucobrassicin is shown.

In vivo labeling of secondary metabolites. A number of known secondary metabolites were detected and identified by MS/MS in the garden cress samples, including sinapic acid derivatives, glucosinolates [47,48] and flavonoids. Figure 3 shows the mass spectra of glucotropaeolin and 4-methoxyglucobrassicin, which were reported to be major representatives of the glucosinolate class in garden cress [49]. Although these compounds are structurally similar, glucotropaeolin likely originates from seeds, while 4-methoxyglucobrassicin demonstrates extensive labeling. The MS/MS spectra of its ^18^O-labeled analogue indicates that the labels are not only in the glucose residue, but also in the sulfonic group.

**Figure 4:**
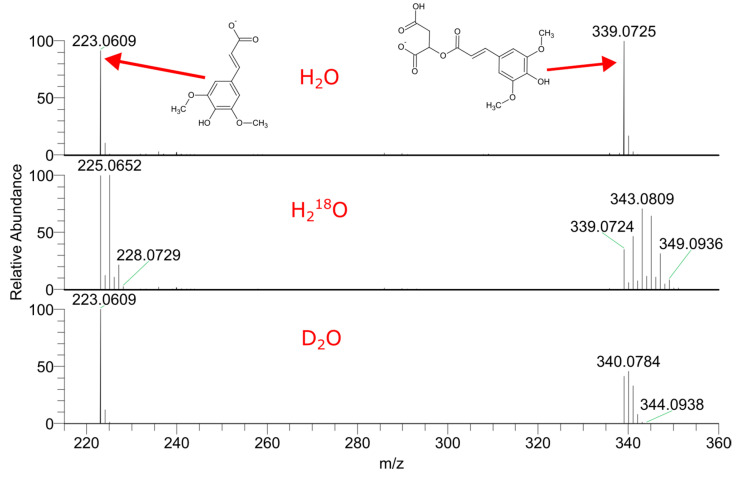
Mass spectra of sinapoyl malate from extracts of unlabeled *Lepidium sativum* seedlings, and of *Lepidium sativum* labeled in vivo with D_2_O and H_2_^18^O.

**Figure 5:**
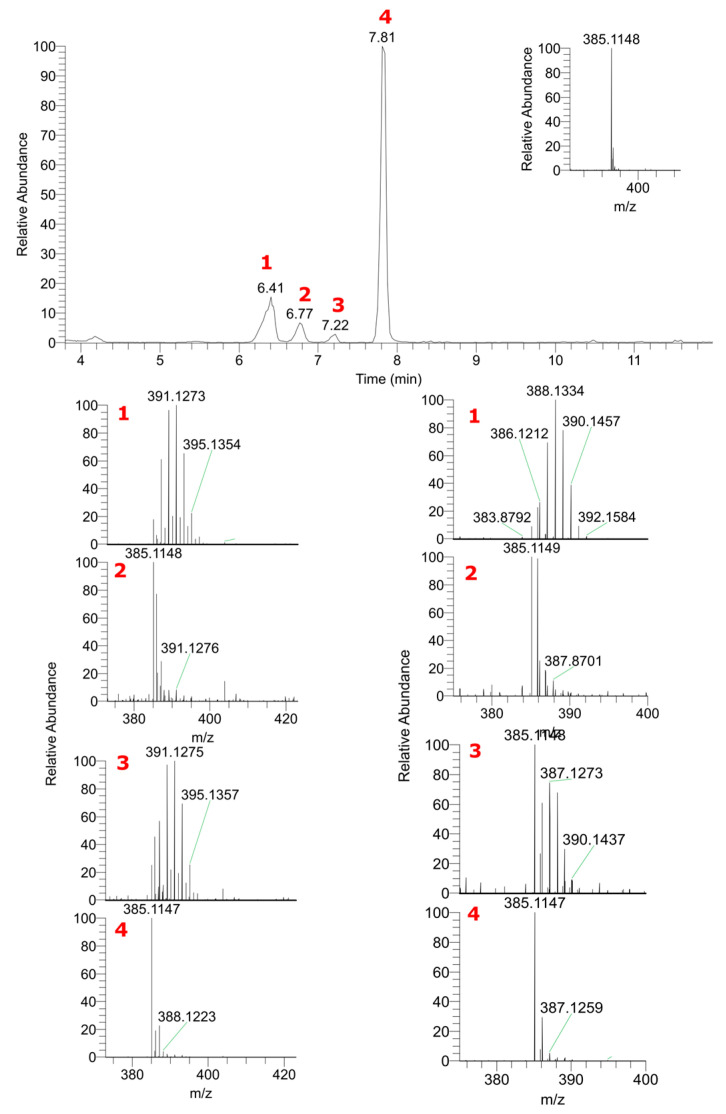
Mass spectra of sinapoyl hexose isomers detected in Lepidium sativum samples in vivo-labeled with D_2_O (left column) and H_2_^18^O (right column).

Figure 4 shows the mass spectra of sinapoyl malate from labeled and non-labeled garden cress. Sinapoyl malate handles three deuterium and six ^18^O labels, and in-ESI fragmentation shows that all the deuterium atoms are in the malate residue, while ^18^O is also in the sinapinic acid fragment. We also did not observe any evidence of sinapic acid biosynthesis when studying the mass spectra of its other derivatives. Thus, sinapine demonstrates no exchanges, and sinapoyl hexose and sinapoyl dihexose carry the labels only in carbohydrate fragments. Figure 5 shows an extracted ion chromatogram for sinapoyl hexose and the mass spectra of all corresponding detected peaks. These peaks are not distinguishable by the fragmentation; however, they demonstrate the differing extents of label incorporation.

## 3. Discussion

Garden cress (Lepidium sativum) is a fast-growing edible herb, and its chemical composition has previously been estimated, with the main focuses on nutritional value [50,51,52], seed oil composition [49,53,54,55,56] or phenolic content [54,57,58,59,60,61,62,63]. Garden cress has been used as a model plant to investigate the metabolism of xenobiotics [64,65,66,67] due its ability for hydroponic cultivation in the laboratory and fast growth. Being well-characterized and easy to handle, Lepidium sativum was chosen as a model plant for experiments with heavy-water-enrichment experiments.

**Figure 6:**
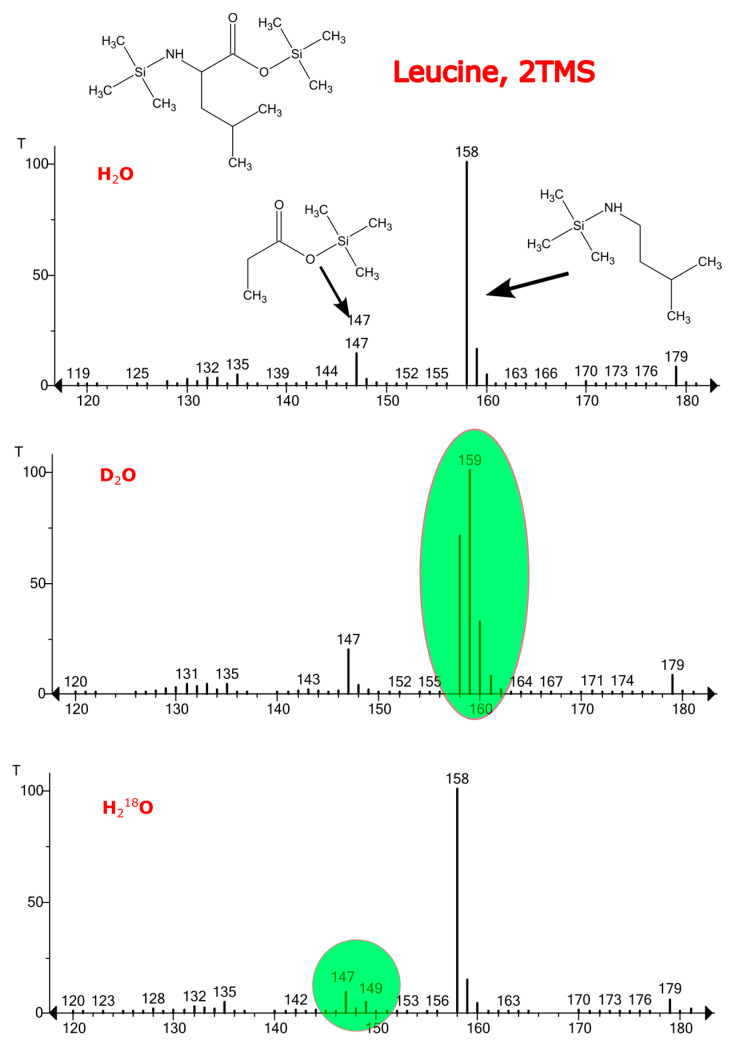
Electron ionization mass spectra of leucine 2TMS derivative from Lepidium sativum sample in vivo-labeled with D_2_O and H_2_^18^O.

A wide range of plant primary and secondary metabolites can be easily labeled in vivo by germinating plants in D_2_O or H_2_^18^O with their reasonable consumption of heavy water. High-resolution mass spectrometry allows the measuring of the number of incorporated heavy atoms by counting the characteristic mass shifts with *m*/*z* 1.0063 and 2.0042 for deuterium and ^18^O, respectively. The main advantage of HRMS is its capability to distinguish between isotopologues; this, in turn, allows us to detect traces of incorporated labels. Low resolution techniques, including conventional GC–MS, also have the capability to detect label incorporation via comparing the isotopic distributions in mass spectra (Figure 6). However, the determination of the replaced atoms by GC–MS is more complicated. First, as most GCs are equipped with low-resolution quadrupole mass analyzers, it is impossible to assign the last peaks of the isotopic distribution with a correct annotation, as they may correspond to external labeling, natural ^13^C isotopes or to the noise/co-eluting compounds. In addition, the resolution of quadrupole instruments may be insufficient to resolve co-eluting compounds. The last problem is related to the electron ionization technique, which causes intensive fragmentation, and in many cases the molecular ion is not registered. As a result, different ions may have different numbers of labels. For example, in the electron ionization mass spectrum of leucine 2TMS derivative, a mass shift of ions with *m*/*z* 158 was detected only in the deuterium-labeled sample, while the peak with *m*/*z* 147 shifted in the H_2_^18^O-labeled sample. The reason for this is that the ion with *m*/*z* 158 does not contain oxygen atoms.

It is well known that the labile hydrogen atoms as well as oxygen atoms from some functional groups can be replaced by their heavy isotopes if a molecule is placed into the heavy isotope-enriched media [32]. This effect should be kept in mind since it may influence the interpretation of the results of in vivo plant labeling. In particular, it may be not clear if the incorporation of the isotope label is the result of biosynthesis or if it is observed due to the isotope exchange equilibrium. As for H/D exchange, this should affect the results to less of an extent because of fast exchange rates in small molecules. As a result, all the exchanged labile atoms will back-exchange during sample preparation and chromatographic separation. Therefore, all the observed deuterium atoms are skeletal (from C-H groups), and their appearance clearly indicates that they were introduced during biosynthesis. On the other hand, ^16^O/^18^O exchange is rather slow, and the introduced label is stable. Previously, we reported ^16^O/^18^O exchange to occur in carbonyl, carboxyl groups and hydroxyl groups in allyl and benzyl positions and in carbohydrates [36,37,38].

Moreover, our results, as shown with the example of malic acid, demonstrate that the ^18^O label is included in metabolites both due to the equilibrium and during biosynthesis. These results are in accordance with the common knowledge regarding the tricarboxylic acid cycle.

**Figure 7:**
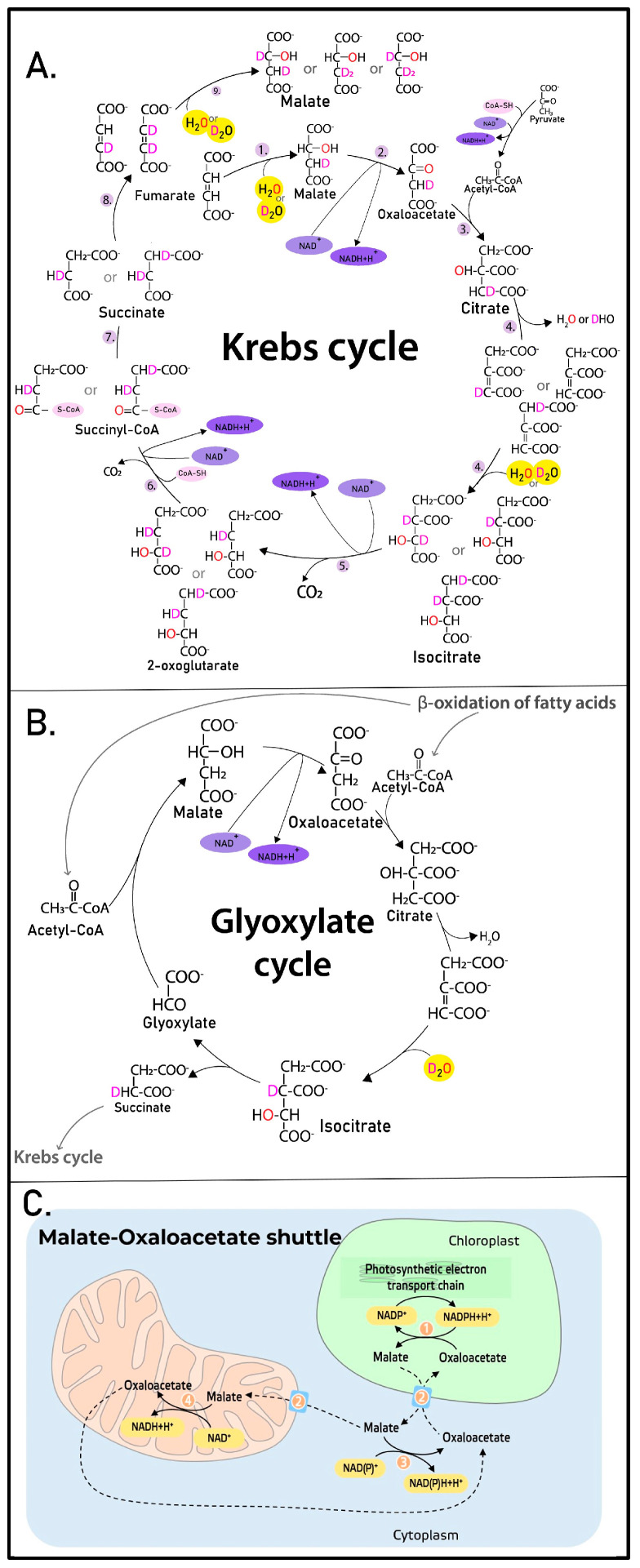
Metabolic pathways and the shuttle in which malate is involved. The inclusion of isotopic labels (red O—heavy oxygen atom ^18^O, pink D—deuterium) is shown. Experimentally, the inclusion of different isotope labels occurred in different experimental systems, not simultaneously. Krebbs cycle (**A**), Glyoxylate cycle (**B**), Malate-Oxaliacetate shuttle (**C**).

It is worth noting that malate in plants can also be produced in the glyoxylate cycle and the malate-oxaloacetate shuttle. However, as shown in Figure 7B,C, malate with three deuterium atoms and one ^18^O cannot be produced using D_2_O and H_2_^18^O as the labeling source. If we follow the transformations of substances in the Krebs cycle (Figure 7A), it can be seen that the inclusion of deuterium and ^18^O can occur in two reactions involving water (Figure 7A, reaction number one—catalyzed by fumarase, reaction number 4—catalyzed by aconitase), and only due to the cyclic nature of the process and the symmetry of some intermediates, deuterium and oxygen can be included in the composition of the malate in the observed MS spectra number. Thus, we assume that the glyoxylate pathway and the malate-oxaloacetate shuttle are not extensively expressed in the studied plants.

**Figure 8:**
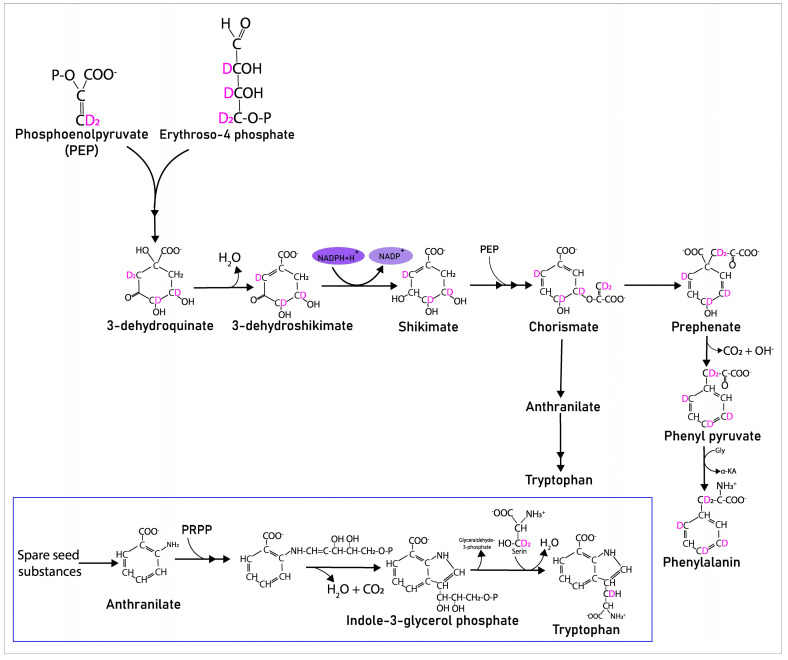
Presumptive inclusion of deuterium into aromatic amino acids during their biosynthesis.

Stable isotope in vivo labeling provides information not only on active metabolic pathways, but also highlights the particular reactions that occur during germination. For example, tryptophan and phenylalanine are produced by plants in the shikimate metabolic pathway [68,69,70]. Since we have observed five deuterium atoms in phenylalanine, and at least some of them are in the aromatic ring, we can assume that phenylalanine was synthesized from the initial precursors of the shikimate biosynthesis pathway (Figure 8) and consider this pathway active. However, tryptophan has only one deuterium atom in its aliphatic chain. This seems strange because if it was synthesized by the shikimate biosynthesis pathway, at least two deuterium atoms should have entered into its aromatic ring from its common precursors with phenylalanine. From this, we can assume that anthranilic acid, formed in the shikimate pathway, was not synthesized during germination. It is likely that anthranilic acid accumulates during the formation of seeds in the female plant before their germination. Anthranilic acid is the predecessor of indole, which condenses with serine to form tryptophan. Although serine was not detected with LC–MS analysis, we could calculate the amount of deuterium from its 3TMS derivative in GC–MS analysis and found that serine has three deuterium labels. Serine reacting with indole results in H_2_O elimination, and in this case at least two deuterium atoms should be found in tryptophan formed from serine-d3. Perhaps, one deuterium is lost during proton migration in the transformations of serine bound to pyridoxal phosphate (a tryptophan synthase coenzyme that performs the final stage of tryptophan synthesis). Here, we have a contradiction that will be the subject of a separate study with additional experiments.

Stable in vivo labeling also allows us to highlight active pathways that lead to the formation of secondary metabolites. The example of sinapoyl malate shows that compounds originating from seeds may interact with newly biosynthesized primary metabolites. Similar observations were made for alkaloid lepidine and semilepidinosides [71]. While lepidines demonstrated no inclusions of heavy isotopes, their glycosides contain both deuterium and ^18^O atoms. For glucosinolates, it was shown that very similar compounds of the same class may be produced at different stages of germination. Thus, there is no evidence of the active synthesis of glucotropaeolin, while for 4-methoxyglucobrassicin we observed labeling in not only the glucose residue but also in the sulfonic group and the aglycone.

The example of sinapoyl hexose demonstrates that, in some cases, stable in vivo labeling may provide an additional dimension for distinguishing between isomeric compounds. In this particular case, four isomers have similar MS/MS spectra and differ only by their retention time. Therefore, for their correct annotation, reference materials are required. At the same time, different labeling extents allow us to make assumptions regarding the structures of metabolites considering their biosynthesis pathways.

## 4. Materials and Methods

### 4.1. Plant Germination and Sample Preparation

Approximately 100 mg of garden cress seeds were sterilized with 70% ethanol, placed into 12 mL glass vials with caps, and 0.3 mL of the corresponding media was added (Day 0). On day 1, 0.9 mL of media was added; on days 4, 7 and 9, we added an additional 0.6 mL of media. Caps were closed to reduce the evaporation of heavy water. Deionized D_2_O:H_2_O (1:1 *v*/*v*) and H_2_^18^O:H_2_O (1:1 *v*/*v*) water mixtures were used as media. On day 12, plants were taken out of the vials and flushed with deionized water to remove seed coatings.

Plants were homogenized with 70% ice-cold methanol (1 mL per 50 mg of fresh plant weight) with a manual homogenizer. Homogenates were centrifuged at 14,000 rpm, and aliquots of 1.5 mL were evaporated to dryness before being re-dissolved in 1 mL of water.

Solid-phase extraction cartridges (Agilent Bond Elut C18, 3 mL, 100 mg sorbent) were conditioned with 2 mL of methanol and equilibrated with 2 mL of 2% formic acid. After application of 1 mL of the sample, the cartridge was flushed with 2 mL of 2% formic acid and the sample was eluted with 2 mL of acetonitrile. The eluate was evaporated to dryness under vacuum at room temperature, and the residue was re-dissolved in 200 μL of deionized water and put into a micro vial for LC–MS analysis.

To prepare samples for GC–MS analysis, SPE eluate was evaporated in a glass vial (nominal volume 2 mL) under vacuum at room temperature. Residues were dissolved in 50 mkl ethyl acetate: BSTFA (with 1% TMCS) (1:1) mixture and incubated at 60 °C for 30 min. Probes were transferred into a glass inserter and placed into a GC–MS autosampler.

### 4.2. LC–MS Analysis

LC–MS analysis was performed on a Q Exactive system (Thermo Scientific Inc., Waltham, MA, USA) coupled with an ACQUITY UPLC system (Waters Corp., Milford, MA, USA). Separation was performed on an ACQUITY UPLC BEH C18 column (2.1 × 100 mm, 1.7 μ) in the following gradient: 5% mobile phase B at 0–5 min, 5% to 75% mobile phase B at 5–25 min, 75% to 100% mobile phase B at 25–26 min, 100% B at 26–33 min, 100% to 5% mobile phase B at 33–35 min, 5% mobile phase B at 35–40 min. The flow rate was set at 0.4 mL min^−1^. Column temperature was maintained at 60 °C. Solutions of 0.1% formic acid in water and acetonitrile were used as mobile phases A and B, respectively. MS analysis was performed in both ESI-positive and negative modes with ESI voltages of 4.5 kV, and 3.5 kV. Data acquisition was performed in DDA mode (Top10) with stepped collision energy (10, 30, 45 V). Resolution and automatic gain control (AGC) were set at 70,000 and 5 × 10^5^ for MS1 and 17,500 and 5 × 10^4^ for MS2. The isolation window was set at 0.4 Da. A representative TIC can be found in Figure S2.

### 4.3. GC–MS Analysis

For analysis, a GC–MS system was used that consisted of a capillary gas chromatograph Agilent 6890 (Agilent, Santa Clara, CA, USA) and an MS detector Agilent 5973N (Agilent, Santa Clara, CA, USA). The column used for analysis was an Agilent HP-5ms column (30 m × 0.25 mm × 0.25 μm). The initial temperature of 60 °C was maintained for 2 min, then ramped up to 290 °C at 10 °C/min and held for 20 min. The injection port temperature was 280 °C, the injection volume was 1 μL and injector was operated in splitless mode. The carrier gas was high-purity helium at a flow rate of 0.9 mL/min. Electron ionization was used with an ionization energy of 70 eV, transmission line temperature of 280 °C, ion source temperature of 230 °C and scan range of 50–550 *m*/*z*. Solvent delay was set to 5 min.

### 4.4. Data Processing

LC–MS data was processed with XCalibur 4.0 and Compound Discoverer 3.3 software (Thermo Scientific, Inc.) and with MS Dial [72,73]. A common routine including peak picking, deconvolution and deisotoping was used to detect components. For primary components annotation, accurate masses and MS/MS spectra of detected components were searched against the mzCloud and MassBank [74] databases with either Compound Discoverer or with MS Dial 4.20 software. Delta *m*/*z* was set at 10 ppm and the annotation score threshold was set at 85. Additionally, we performed a targeted search of compounds that were previously reported to be found in *Lepidium sativum*. These compounds were searched by accurate mass and the annotation was confirmed via the manual interpretation of MS/MS spectra. An example interpretation is presented in Figure S1. All the annotations were checked manually to increase the confidence in the results.

GC–MS data were analyzed with ChemStation Data Analysis E.02.02.1431 software. Components from GC–MS data were finally determined using AMDIS software and annotated with a library search against the NIST20 library with a match factor threshold of 85. The Top-1 candidate is reported.

### 4.5. Data Availability

Raw data are available via the following link: https://drive.google.com/file/d/1j5mpxW5hgdQp40CcKsB3XuQ6UI-KnJAU/view?usp=sharing (accessed 31 August 2023).

## 5. Conclusions

Stable isotope in vivo labeling of plants with D_2_O and H_2_^18^O followed by a combination of LC–HRMS and conventional GC–MS analysis may provide new insights into plant metabolic pathways and aid metabolite annotation. Varying the design of in vivo labeling experiments may be a subject of future studies to investigate metabolism at particular stages of germination or to evaluate the effects of external conditions on the metabolome and metabolic pathways.

## Figures and Tables

**Table 1:** Compounds annotated in *Lepidium sativum* extracts after in vivo labeling.

Compound	Formula	Number of Detected D	Number of Detected ^18^O
Arginine	C_6_H_14_N_4_O_2_	0	2
Asparagine	C_4_H_8_N_2_O_3_	3	3
Aspartate	C_4_H_7_NO_4_	3	4
Glutamine	C_5_H_10_N_2_O_3_	5	3
Histidine	C_6_H_9_N_3_O_2_	3	2
Isoleucine	C_6_H_13_NO_2_	6	2
Leucine	C_6_H_13_NO_2_	6	2
Lysine	C_6_H_14_N_2_O_2_	1	2
Phenylalanine	C_9_H_11_NO_2_	5	2
Threonine	C_4_H_9_NO_3_	2	3
Tryptophan	C_11_H_12_N_2_O_2_	1	2
Tyrosine	C_9_H_11_NO_3_	4	3
Valine	C_5_H_11_NO_2_	5	2
Proline	C_5_H_9_NO_2_	1	2
Methionine	C_5_H_11_NO_2_S	3	2
Pyroglutamic acid	C_5_H_7_NO_3_	5	2
Malic acid	C_4_H_6_O_5_	3	2
Fumaric acid	C_4_H_4_O_4_	2	2
Succinic acid	C_4_H_6_O_4_	4	2
Benzoic acid	C_7_H_6_O_2_	0	2
Glucose-6-phosphate	C_6_H_13_O_9_P	5	2
Betaine	C_5_H_11_NO_2_	6	2
Phosphocholine	C_5_H_15_NO_4_P+	0	2
p-Coumaric acid	C_9_H_8_O_3_	2	2
Acetyl-L-Carnitine	C_9_H_17_NO_4_	ND	2
Glucotropaeolin	C_14_H_19_NO_9_S_2_	0	2
4-methoxyglucobrassicin	C_17_H_22_N_2_O_10_S_2_	9	2
Sinapoyl malate	C_15_H_16_O_9_	3	2
Sinapine	C_16_H_24_NO_5_	0	2
Kaempferol rhamnose-hexoside	C_33_H_40_O_20_	0	2
Semilepidinoside A	C_16_H_20_N_2_O_6_	6	2
Semilepidinoside B	C_17_H_22_N_2_O_7_	8	2
Lepidine A/C	C_21_H_20_N_4_O_2_	0	0
Lepidine B/D/E/F	C_20_H_18_N_4_O_2_	0	0

## Data Availability

All data can be requested from authors.

## Supplementary Materials

The supporting information can be downloaded at: https://www.mdpi.com/article/10.3390/ijms242015396/s1.
