# Supplementary material for: Investigating the Metabolism of Plants Germinated in Heavy Water, D2O, and H218O-Enriched Media Using High-Resolution Mass Spectrometry
Source: Int J Mol Sci. 2023 Oct 20;24(20):15396. doi: 10.3390/ijms242015396 (PMC10607710; doi:10.3390/ijms242015396)
Supplement: Supplementary file 1 [file ijms-24-15396-s001.zip › ijms-2574177-supplementary.pdf]

Table S1. Compounds annotated in garden cress with GC-MS

RT(min)	Chemical Name
6.84	Ethanolamine, 2TMS derivative
7.34	Lactic Acid, 2TMS derivative
7.98	L-Alanine, 2TMS derivative
8.24	Glycine, di-TMS
9.69	L-Valine, 2TMS derivative
10.07	Urea, 2TMS derivative
10.25	Benzoic Acid, TMS derivative
10.50	L-Leucine, 2TMS derivative
10.57	Glycerol, 3TMS derivative
10.83	L-Proline, 2TMS derivative
10.96	2-Butenedioic acid, (Z)-, 2TMS derivative
11.04	Butanedioic acid, 2TMS derivative
11.35	Glyceric acid, 3TMS derivative
11.44	Uracil, 2TMS derivative
11.49	2-Butenedioic acid, (E)-, 2TMS derivative
11.75	Serine, 3TMS derivative
12.11	L-Threonine, 3TMS derivative
13.04	Dihydroxymalonic acid, 4TMS derivative
13.40	Malic acid, 3TMS derivative
13.82	L-Aspartic acid, 3TMS derivative
13.83	L-Aspartic acid, 3TMS derivative
13.83	L-5-Oxoproline, 2TMS
13.83	L-Aspartic acid, 3TMS derivative
14.93	L-Glutamic acid, 3TMS derivative
15.00	Phenylalanine, 2TMS derivative
15.10	Arabinofuranose, 1,2,3,5-tetrakis-O-(trimethylsilyl)-
15.54	Homoserine, 4-imino-N,O-bis(trimethylsilyl)-, trimethylsilyl ester
16.03	D-Xylose, 4TMS derivative
16.61	Phosphoric acid, bis(trimethylsilyl) 2,3-bis[(trimethylsilyl)oxy]propyl ester
16.86	2-Keto-l-gluconic acid, penta(O-trimethylsilyl)-
17.10	D-(-)-Fructofuranose, pentakis(trimethylsilyl) ether (isomer 1)
17.19	Citric acid, 4TMS derivative
17.50	D-(+)-Talofuranose, pentakis(trimethylsilyl) ether (isomer 2)
17.90	Syringic acid, 2TMS derivative
18.00	.beta.-D-(+)-Mannopyranose, 5TMS derivative
18.00	2,3,4,5,6-Pentahydroxyhexanal, 5TMS
18.24	2,4-Dihydroxybenzaldehyde, 2TMS derivative
18.43	Ethyl .alpha.-D-glucopyranoside, 4TMS derivative
18.86	.beta.-D-Glucopyranose, 5TMS derivative
19.12	D-Gluconic acid, 6TMS
19.21	Palmitic Acid, TMS derivative
19.53	Glucaric acid, 6TMS derivative
19.70	9,12-Octadecadienoic acid, methyl ester
20.71	9,12-Octadecadienoic acid (Z,Z)-, TMS derivative
20.76	11-Octadecenoic acid, (Z)-, TMS derivative
20.78	.alpha.-Linolenic acid, TMS derivative
20.87	D-Glucose, 4-O-[.beta.-D-glucopyranosyl], 8TMS
20.98	Stearic acid, TMS derivative

21.06	Sinapinic acid, 2TMS derivative
21.16	Carbonic acid, monoamide, N-(2-pentyl)-N-hexadecyl-, propargyl ester
21.90	2-O-Glycerol-.alpha.-d-galactopyranoside, hexa-TMS
22.41	11-Eicosenoic acid, (Z)-, TMS derivative
22.62	Arachidic acid, TMS derivative
23.29	2-Phenylethyl .beta.-D-glucopyranoside, 4TMS derivative
23.80	1-Monopalmitin, 2TMS derivative
24.30	.alpha.-D-glucopyranose, 1-O-(3-O-(2-methylbutanoyl)-.alpha.-D glucopyranosyl), 7TMS derivative
24.56	Sucrose, 8TMS derivative
25.08	1-Monooleoylglycerol, 2TMS derivative
25.14	1-Linolenoylglycerol, 2TMS derivative
25.41	D-Trehalose 8TMS
28.37	3,5,7-Trihydroxy-2-(4-hydroxyphenyl)-4H-chromen-4-one, 3TMS

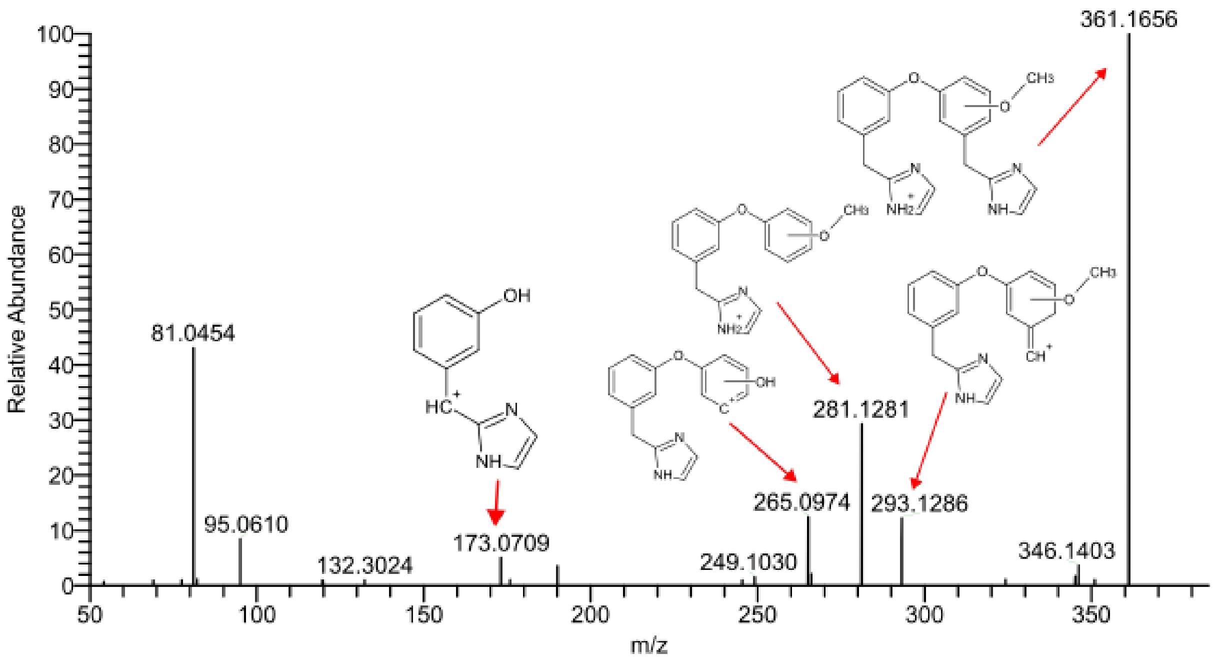

Figure S1. Manual interpretation of MS/MS spectrum on the example of Lepidine A/C.

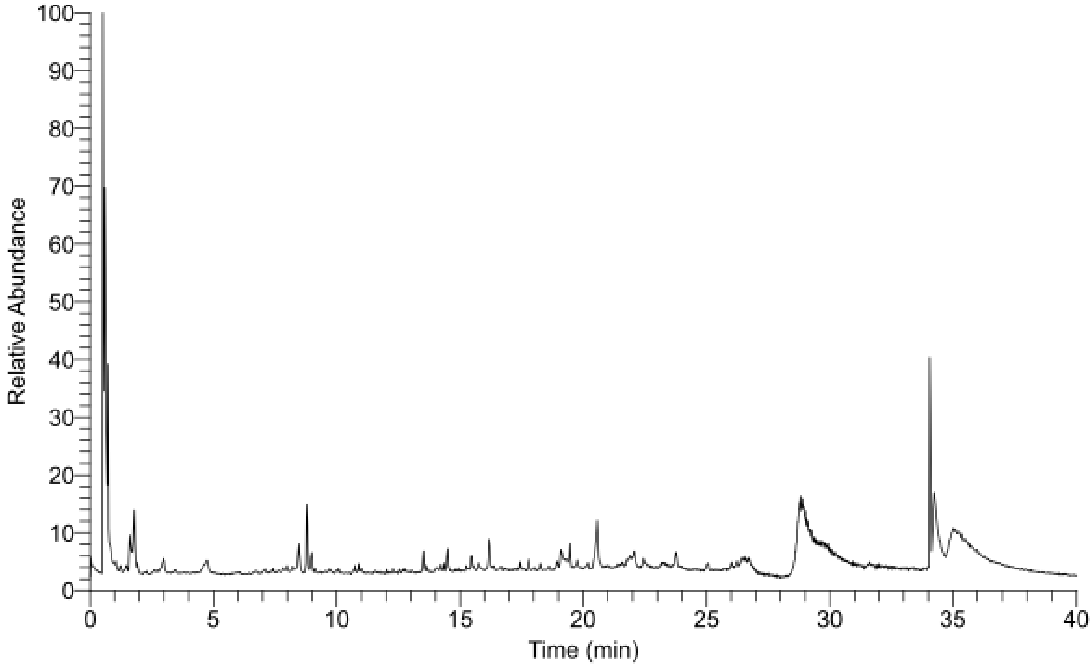

Figure S2. A representative negative ionization mode TIC chromatogram of an extract of Lepidium sativum germinated on H_2O.
